# Bond Capability of Universal Adhesive Systems to Dentin in Self-etch Mode after Short-term Storage and Cyclic Loading

**DOI:** 10.2174/1874210601711010276

**Published:** 2017-06-30

**Authors:** Daniele Morosini Costa, Deise Caren Somacal, Gilberto Antonio Borges, Ana Maria Spohr

**Affiliations:** 1 Department of Restorative Dentistry, School of Dentistry, Pontifical Catholic University of Rio Grande do Sul, Porto Alegre, Brazil.; 2 Department of Restorative Dentistry, Uberaba University, Uberaba, Brazil.; 3 Department of Dental Materials, School of Dentistry, Pontifical Catholic University of Rio Grande do Sul, Porto Alegre, Brazil.

**Keywords:** Bond strength, Dentin, Universal adhesives, Composite resin, Bovine teeth

## Abstract

**Objective:**

The aim was to evaluate, *in vitro*, the tensile bond strength to dentin of Scotchbond Universal (SU), All-Bond Universal (AU) and One Coat 7 Universal (OC7) adhesives applied in self-etch mode, after 24 h of storage and after 500,000 loading cycles, using Clearfil SE Bond (SE) as a control.

**Materials and Methods:**

The adhesives were applied on the dentin of bovine teeth, followed by the application of a composite resin. Thirty specimens were obtained for each adhesive. Half of the specimens were submitted to cyclic loading for 500,000 cycles. All specimens were submitted to a tensile bond strength test in a universal testing machine at a crosshead speed of 0.5 mm/minute.

**Results:**

According to two-way ANOVA and Tukey’s test (α=5%), the interaction between the adhesive and cyclic loading factors was significant (p=0.001). The means followed by the same letter represent no significant difference in the bond strength (MPa) after 24 h: OC7=7.86^A^ (±2.90), SU=6.78^AB^ (±2.03), AU=5.61^BC^ (±2.32), and SE=3.53^C^ (±1.89). After cyclic loading, SE, SU and AU maintained bond strength comparable to 24 h period. There was a significant decrease only for OC7.

**Conclusion:**

SU, AU and OC7 had bond strength to dentin comparable to that of SE. Only OC7 had decreased bond strength to dentin after cyclic loading.

## INTRODUCTION

Self-etch adhesive systems differ technically from etch and rinse adhesive systems due to the elimination of 35% phosphoric acid etching and, consequently, elimination of the step of water removal after etching [1]. Self-etch adhesive systems present acidic monomers that are responsible for enamel and dentin demineralization [2].

The acidic monomers of self-etch adhesive systems may be derivatives of carboxylic acid groups (4-META) or phosphate acid groups (phenyl-P, 10-MDP, PENTA) [3]. Self-etch adhesive systems contain water that serves to ionize the acidic monomers, enabling them to demineralize the smear layer and the underlying dentin to form the hybrid layer [4, 5]. These adhesive systems can be classified according to their pH as strong (pH ≤1), intermediate (pH = 1.5), or mild (pH >2) [6]. Depending on the pH, the smear layer and smear plugs are not dissolved completely and the thickness of the hybrid layer varies according to the pH [1, 7]. Usually, self-etch adhesive systems do not provide a selective demineralization of the enamel similar to that with 35% phosphoric acid [8]. Thus, selective enamel etching in a separate step with 35% phosphoric acid has been recommended prior to application of the self-etching adhesive system [9].

Several companies have launched self-etch adhesive systems. However, Clearfil SE Bond has been shown to be one of the most reliable self-etch adhesive systems, and it presents high bond strength values to dentin and is considered the gold standard for this category of adhesive systems [10, 11].

Recently, universal adhesive systems were launched with the aim of technical simplification. These adhesive systems are classified as “universal” because they may be used as self-etch adhesives, etch-and-rinse adhesives, or as self-etch adhesives on dentin and etch-and-rinse adhesives on enamel (selective enamel etching). In addition, these adhesives can be applied on the surface of different restorative materials [12]. Three representatives of this category are the Scotchbond Universal, All-Bond Universal and One Coat 7 Universal.

Regardless of the commercial presentation and application technique, it is important that universal adhesive systems are comparable to or have better performance than the gold standard adhesive system. Regarding the application mode, studies have shown that self-etch mode improved the bonding effectiveness of universal adhesives on dentin [13, 14].

 Based on this finding, the aim of the current study was to evaluate, *in vitro*, the bond strength to dentin of Scotchbond Universal, All Bond Universal and One Coat 7 Universal, applied in self-etch mode, after 24 h of storage and after 500,000 loading cycles. This study was conducted with the null hypothesis that no significant difference in bond strength exists between the universal adhesive systems and the gold standard adhesive system.

## MATERIALS AND METHODS

### Tooth Preparation

One hundred and twenty permanent bovine incisors, extracted at the age of 2 years, were selected. The teeth were cleaned of gross debris and stored in distilled water at 4°C. The water was changed every week and the teeth were used within six months.

The crowns were sectioned at the superior and inferior thirds to obtain 10 mm-high coronary portions. The coronal portions were placed with the buccal surface against a glass plate with wax. A metallic cylindrical device was used to embed the teeth. The device was placed on the glass plate so that the dental surface was centered. Then, self-cured acrylic resin (Jet Clássico, São Paulo, SP, Brazil) was used to fill the metallic cylindrical device.

 In a lathe under water spray (Nardini - ND 250 BE, Americana, SP, Brazil), 2 mm of acrylic resin and tooth were removed, exposing the dentin. The dentin surfaces were hand-polished with wet 400 and 600 grit silicon carbide abrasive paper (Carborundum Abrasives, Recife, PE, Brazil) and rinsed with water for 15 s, and the excess water was removed by air drying.

### Bonding Procedures

The embedded teeth were randomly assigned to four groups (n=30) for bonding with the adhesive systems (Table **1**) that were applied in self-etch mode.

### According to the Manufacturers.

Group 1- Clearfil SE Bond (control): The self-etching primer was applied to the dentin using a microbrush and was left in place for 30 s. Excess solvent was removed by air drying for 5 s. The bond was applied using a microbrush, followed by gentle air drying for 5 s and light curing for 20 s with a Radii Cal curing unit (SDI, Vic., Australia) with light intensity of 1.000 mW/cm^2^.

Group 2- Scotchbond Universal: The adhesive was applied to the dentin with a microbrush and scrubbed for 20 s, followed by gentle air drying for 5 s and light curing for 10 s.

Group 3- All-Bond Universal: The adhesive was applied to the dentin with a microbrush and scrubbed for 20 s, followed by gentle air drying for 5 s and light curing for 10 s.

Group 4- One Coat 7 Universal: The adhesive was applied to the dentin with a microbrush and scrubbed for 20 s, followed by gentle air drying for 5 s and light curing for 10 s.

A metallic split cylinder, 4 mm high with an orifice 3 mm in diameter at the bottom and 5 mm in diameter at the top, was placed against the specimen so that the orifice was over the treated dentin. Composite resin Z250 (3M, St. Paul. MN, USA) was inserted in the interior of the orifice in two increments to form an inverted cone of composite resin which provided a grip for the clutch used in the tensile bond test. Each increment was light cured for 40 s.

The specimens were stored in distilled water at 37°C for 24 h. Half of the specimens in each group (n=15) were submitted to tensile bond strength test, and the other 15 specimens were tested after cyclic loading (Erios ER-11000, Erios, São Paulo, SP, Brazil) at 50 N for 500,000 cycles at 1 Hz in distilled water at 37 ^o^ C.

### Tensile Bond Strength Testing

The tensile bond strength test was performed in a universal testing machine (EMIC DL-2000, São José dos Pinhais, PR, Brazil) at a crosshead speed of 0.5 mm/min. The tensile bond strength values, in MPa, were calculated from the peak load at failure divided by the specimen surface area.

After the tensile bond strength tests, the fractured surfaces of the specimens were visually examined with a stereomicroscope (Olympus Corp., Tokyo, Japan) at 20X to classify the type of failure that occurred during the debonding procedure. The failures were classified as follows: a) adhesive (rupture in the interface between the dentin and the adhesive system); b) cohesive in dentin (dentin substrate failure); c) cohesive in composite resin (composite resin failure); or d) mixed (adhesive and cohesive failure in the dentin or composite resin) [15].

### Statistical Analysis

Statistical analysis was performed by applying a two-way analysis of variance (adhesive system x cyclic loading) followed by Tukey’s post hoc test at a 95% confidence level.

## RESULTS

Two-way ANOVA analysis revealed that the interaction effect (adhesive system x cyclic loading) was significant (*p*<0.05). According to Tukey’s test, without cyclic loading, higher tensile bond strength was obtained with One Coat 7 Universal (7.86 MPa), though not significantly different from that of Scotchbond Universal (6.78 MPa) (*p*>0.05). All-Bond Universal had an intermediate value (5.61 MPa), though not significantly different from those of Scotchbond Universal and Clearfil SE Bond (3.53 MPa). With cyclic loading, there was no significant difference in tensile bond strength between the adhesive systems (*p*>0.05). One Coat 7 Universal was the only adhesive system that had significantly decreased tensile bond strength after cyclic loading (4.26 MPa) (*p*<0.05) (Table **2**).

There was a predominance of adhesive failures in almost all groups, especially for Clearfil SE Bond. Cohesive failures in the composite resin occurred in all groups and were the second most predominant failure in the study. There were mixed failures, except with Clearfil SE Bond without cyclic loading. Cohesive failure in the dentin occurred in only two specimens (Table **3**).

## DISCUSSION

The results of the present study are positive, as the universal adhesive systems tested obtained bond strengths greater than or comparable to that of Clearfil SE Bond, which was considered the control. Based on the results, the null hypothesis was rejected.

According to the manufacturers, the three universal adhesive systems tested in the present study can be applied in etch and rinse mode or self etched mode on dentin. Studies have compared the bond strength of universal adhesives to dentin using the etch and rinse or the self-etch mode, and similar bond strength values were observed for each adhesive system regardless of the application mode [16-19]. However, there is presently a preference for the self-etch adhesive system on dentin due to shallower demineralization compared to 35% phosphoric acid [20, 21] and the elimination of the water removal step after etching with phosphoric acid; this is one of the most critical steps during etch-and-rinse adhesive system application [22]. In addition, 35% phosphoric acid removes calcium from the dentin surface, leaving a network of collagen fibers surrounded by water [1]. The removal of calcium from the dentin surface might avoid any potential ionic bonding between calcium and the phosphate and/or carboxylate groups present in the adhesive, decreasing the bond capability to dentin, especially after aging [13, 14]. Thus, the present study tested the universal adhesive systems in self-etch mode.

The chemical composition of the adhesive system directly influences the bonding ability [23]. All of the adhesive systems tested include the 10-methacryloyloxydecyl dihydrogen phosphate monomer (10-MDP). The 10-MDP monomer provides acidity and, consequently, the capability to etch the dentin surface. During the demineralization promoted by the acidic monomer, other substances present in the adhesive infiltrate into the demineralized dentin [1, 3]. Another important factor related to 10-MDP is that it has the ability to chemically bond to the hydroxyapatite in dentin and enamel, favoring the bond to dental tissue [3, 24, 25]. In self-etch mode, the residual hydroxyapatite that remains around the collagen fibrils serves as a receptor for chemical interaction with 10-MDP and subsequently contributes to adhesive performance [3].

Scotchbond Universal includes a polyalkenoic acid copolymer which chemically bonds to the calcium in hydroxyapatite [26]. More than 50% of the carboxyl groups in the polyalkenoic acid copolymer are able to bond to hydroxyapatite. Carboxylic groups replace phosphate ions on the substrate creating ionic bonds with calcium [27]. There is a high chance that the presence of polyalkenoic acid copolymer leads to higher bond stability between the dentin and the adhesive during the 6 months of storage [14]. It is likely that the presence of polyalkenoic acid copolymer favors additional bonding of Scotchbond Universal to dentin. The study by Perdigão *et al.* [28] also tested the bond strengths of Scotchbond Universal and Clearfil SE Bond to dentin and the values were higher for Scotchbond Universal.

Adhesive systems can be classified in relation to the pH as strong, intermediate, and mild [6]. The pH of the adhesive system determines the ability of the materials to demineralize dentin and enamel [1, 7]. All-Bond Universal (pH=3.2), Scotchbond Universal (pH=2.7), One Coat 7 Universal (pH= 2.0-2.8) and Clearfil SE Bond (pH=2) are classified as mild adhesive systems. Some newer one-step self-etch adhesive systems with mild acidity have shown improved performance in comparison with strong one-step self-etch adhesives on dentin [29]. However, a 36-month clinical trial evaluating Scotchbond Universal showed signs of degradation and more restorations were lost when this universal adhesive was applied in self-etch mode compared to the selective enamel etching technique [30].

In the present study, cyclic loading was applied to half of the specimens as an *in vitro* aging method to simulate masticatory loads. The specimens were submitted to cyclic loading at 50 N for 500,000 cycles, simulating two years of function [31]. During cyclic loading, the specimens were immersed in distilled water at 37°C to reproduce the humidity conditions of the oral cavity. One Coat 7 Universal was the only adhesive system that had a significant decrease in bond strength after cyclic loading. For the other three adhesive systems, the cyclic loading did not influence the bond strength values. An explanation for this finding may be related to the composition of the adhesives. Although manufacturers do not specify the precise percentage of each component present in the adhesives, it is possible that the presence of different percentages of the 10-MDP monomer can make the adhesive more or less vulnerable to the degradation process. The bond of 10-MDP to calcium in hydroxyapatite creates a salt (10-MDP-Ca) that protects against hydrolysis [32] because it is a hydrolytically stable salt [24], favoring maintenance of bond strength values.

The hydrolytic degradation process is a reality in adhesive systems. The literature has shown that single-bottle adhesive systems have higher hydrolytic degradation compared to three-step etch-and-rinse adhesive systems or two step self-etch adhesive systems such as Clearfil SE Bond. An explanation for the higher degradation is that single-bottle adhesive systems contain water and more hydrophilic monomers in their composition [33], in addition to their lack of a hydrophobic adhesive layer [34]. The bonded interfaces of one-bottle adhesive systems behave as semi-permeable membranes that allow the movement of water across them and favor hydrolytic degradation [35]. However, Scotchbond Universal and All-Bond Universal are one-bottle simplified adhesives and there was no decrease in their bond strength after cyclic loading underwater. The possibility that the duration of the cyclic loading performed in the present study was not long enough to cause a significant decrease in bond strength cannot be disregarded. Probably, a long-term water storage, or a combination of aging methodologies could have decreased the bond strength [36]. However, water has been shown to pass through adhesives that do not contain a hydrophobic layer in the initial minutes after application to dentin [37].

The analysis of failures is a mandatory methodology when bond strength tests are performed. This analysis aims to determine where the rupture occurs in the tensile test because the location of the rupture corresponds to the value obtained in megapascals. When the rupture occurs cohesively in the composite resin cone, the value obtained corresponds to the cohesive strength of the composite resin. However, in the evaluation of adhesive systems on substrates, the objective is to study the bond of the adhesive with the substrate, either on dentin or on enamel, and not in other regions such as cohesion in the dentin or composite resin. Of the 15 specimens evaluated in each group, most failures were classified as adhesive and/or mixed failures (adhesive failure and cohesive failure in the composite resin cone). Therefore, in most specimens, the adhesive interface was evaluated. Moreover, the cyclic loading did not cause a significant change in the failure mode, showing that this aging methodology did not influence the bond capacity of the adhesive systems evaluated.

Both permanent and deciduous bovine incisors have been used to test the bond strength of adhesive systems to enamel and dentin. Deciduous enamel showed lower bond strength than permanent enamel [38], while the bond strengths on the deciduous dentin were higher or similar than those on the permanent dentin, depending on the adhesive system and thermal cycled application [39]. Regardless the differences between deciduous and permanent bovine incisors, permanent incisors are most frequently used in tests of adhesion between restorative materials and dental hard tissue as a substitute for human teeth [40]. Due to this reason, permanent bovine incisors were selected in the present study, which present a resemblance to human teeth [41] and are easily obtained in large amounts from registered slaughterhouses.

The simplification of the universal adhesive systems significantly facilitates their use in clinical practice and decreases the possibility of application errors due to the reduction of application steps. However, longitudinal clinical studies are fundamental to verify the performance of the universal adhesive systems.

## CONCLUSION

The results of the current study demonstrate that the Scotchbond Universal, All-Bond Universal and One Coat 7 Universal, applied in self-etch mode, obtained bond strength to dentin similar to or superior to that of the Clearfil SE Bond. One Coat 7 Universal was the only adhesive system that presented a decrease in bond strength after cyclic loading.

## Figures and Tables

**Table 1:** Composition of the adhesive systems.

Adhesive System	Composition	Batch Number	Manufacturer
All-Bond Universal	Adhesive: 10-MDP, Bis-GMA, HEMA, ethanol, water, initiators	1400007770	Bisco, Schaumburg, IL, USA
Scotchbond Universal	Adhesive: 10-MDP, phosphate monomer, dimethacrylate resins, HEMA, methacrylate-modified polyalkenoic acid copolymer, filler, ethanol, water, initiators, silane	579967	3M-ESPE, St. Paul, MN, USA
Clearfil SE Bond	Primer:10-MDP, HEMA, camphorquinone, hydrophilic dimethacrylate, water Bond: 10-MDP, Bis-GMA, HEMA, camphorquinone, hydrophobic dimethacrylate, N,N-diethanol ptoluidine bond, colloidal silica	051539	Kuraray, Kurashiki, Okayama, Japan
One Coat 7 Universal	Methacrylates including 10-MDP, photoinitiators, ethanol, water	F96836	Coltène, Cuyahoga Falls, OH, USA

**Table 2:** Tensile bond strength means (MPa), standard deviations (±SD) and minimum and maximum values for the groups without and with cyclic loading.

	Group 1 – Clearfil SE Bond	Group 2 – Scotchbond Universal	Group 3 – All-Bond Universal	Group 4 – One Coat 7 Universal
Without cyclic loading	3.53 ^Ca^ (±1.89) Minimum (1.71) Maximum (6.21)	6.78^ABa^ (±2.03) Minimum (4.51) Maximum (11.93)	5.61 ^BCa^ (±2.32) Minimum (2.96) Maximum (9.65)	7.86 ^Aa^ (±2.90) Minimum (3.77) Maximum (11.75)
With cyclic loading	4.70 ^Aa^ (±2.50) Minimum (2.63) Maximum (9.36)	6.05 ^Aa^ (±1.44) Minimum (4.35) Maximum (9.78)	5.89 ^Aa^ (±2.11) Minimum (3.49) Maximum (9.36)	4.26 ^Ab^ (±1.20) Minimum (2.75) Maximum (6.40)

* The means followed by the same capital letter in lines and by the same lowercase letter in columns are not significantly different according to Tukey’s test (α=0.05).

**Table 3:** Failure mode analysis.

Group / Failure Mode	Adhesive	Cohesive in Composite Resin	Cohesive in Dentin	Mixed (Adhesive and Cohesive in Composite Resin)
Clearfil SE Bond without cyclic loading	13	2		
Clearfil SE Bond with cyclic loading	9	4		2
Scotchbond Universal without cyclic loading	8	5		2
Scotchbond Universal with cyclic loading	9	4		2
All-Bond Universal without cyclic loading	5	4		6
All-Bond Universal with cyclic loading	6	4	1	4
One Coat 7 Universal without cyclic loading	5	3	1	6
One Coat 7 Universal with cyclic loading	8	6		1
